# Erratum: Huh et al., “Time Course of Alterations in Adult Spinal Motoneuron Properties in the SOD1(G93A) Mouse Model of ALS”

**DOI:** 10.1523/ENEURO.0370-23.2023

**Published:** 2023-10-12

In the article “Time Course of Alterations in Adult Spinal Motoneuron Properties in the SOD1(G93A) Mouse Model of ALS,” by Seoan Huh, Charles J. Heckman, and Marin Manuel, which was published online on February 25, 2021, Figure 8 appeared incorrectly. A sign error occurred when calculating the effect size of the results by Martin et al. (2013), resulting in errors to the embryonic time point presented in the figure. The corrected figure appears below. This correction does not affect the conclusions of the article.

**Figure 8.**
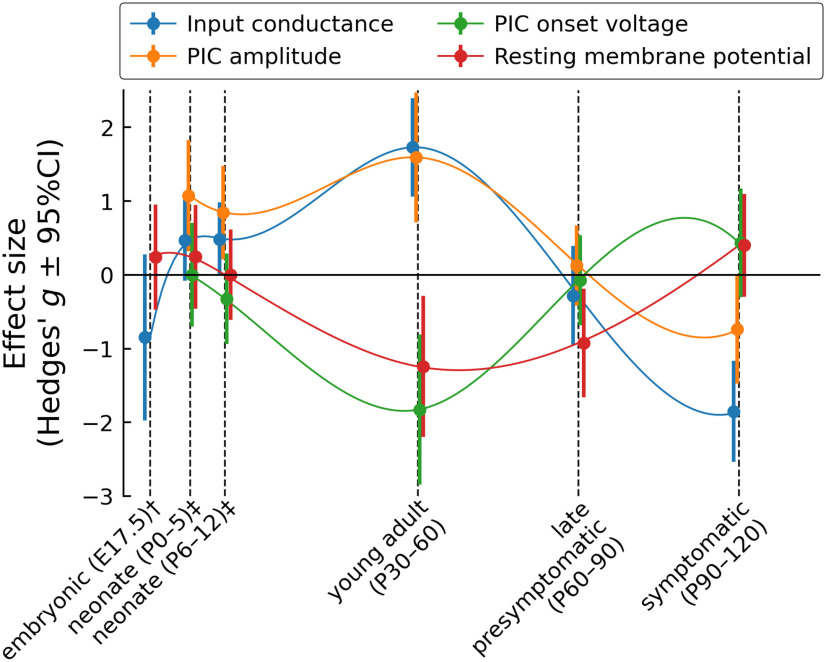
Summary of the changes in motoneuron properties over time. Schematic representation of the changes in four key electrophysiological properties over time. The dots represent the effect size (Hedges’ *g*) and the vertical bars show the 95%CI around *g*. The thin lines are cubic splines interpolation of the data over time. The points have been slightly staggered so that the vertical bars do not occlude each other. †Data from embryonic motoneurons are from Martin et al. (2013). These authors did not measure PICs in embryonic motoneurons. Kuo et al. (2004) did measure PICs, but their embryonic motoneurons were cultured for 10–30 d *in vitro*, and their development stage is therefore uncertain. ‡Data from neonates (P0–P5 and P6–P12) are from Quinlan et al. (2011).

